# DETERMINANTS OF GENERAL WELL-BEING IN BLACK MALES WITH CHRONIC ILLNESS

**DOI:** 10.1093/geroni/igz038.1451

**Published:** 2019-11-08

**Authors:** Darlingtina Atakere

**Affiliations:** University of Kansas, Lawrence, Kansas, United States

## Abstract

Over the last decades, considerable attention has been directed towards examining the well-being of people living with chronic illness. The presence of one or more chronic illnesses challenges their quality of life and general well-being, thus, impacting their abilities to function physically, psychologically, and socially. I investigated reports of general well-being in Black males with chronic illness(es) in a sample of N=242 participants. The males were aged 35–63 and identified as Black/African American males. The participants responded to items assessing general well-being; ethnic identity; self-esteem; active coping; the presence of chronic illness(es); and additional demographic, social and ecological characteristics. Analyses of responses indicated that marital status, ethnic identity, self-esteem are significant determinants of general well-being in Black males with chronic illness(es). Data further showed active coping to be negatively correlated with well-being. I discuss the implications of results for the understanding of health outcomes among this marginalized population.

